# Depression treatment in Germany – using claims data to compare a collaborative mental health care program to the general practitioner program and usual care in terms of guideline adherence and need-oriented access to psychotherapy

**DOI:** 10.1186/s12888-020-02995-1

**Published:** 2020-12-14

**Authors:** Alexander Engels, Hans-Helmut König, Julia Luise Magaard, Martin Härter, Sabine Hawighorst-Knapstein, Ariane Chaudhuri, Christian Brettschneider

**Affiliations:** 1 Department of Health Economics and Health Services Research, Center for Psychosocial Medicine, University Medical Center Hamburg-Eppendorf, Martinistr. 52, Building W37, 20246 Hamburg, Germany; 2 Department of Medical Psychology, Center for Psychosocial Medicine, University Medical Center Hamburg-Eppendorf, Hamburg, Germany; 3 AOK Baden-Württemberg, Stuttgart, Germany

**Keywords:** Major depressive disorder, Guideline adherence, Antidepressants, Psychotherapy, Waiting times

## Abstract

**Background:**

Societies strive for fast-delivered, evidence-based and need-oriented depression treatment within budget constraints. To explore potential improvements, selective contracts can be implemented. Here, we evaluate if the German collaborative psychiatry-neurology-psychotherapy contract (PNP), which extends the gatekeeping-based general practitioner (GP) program, improved guideline adherence or need-oriented and timely access to psychotherapy compared to usual care (UC).

**Methods:**

We conducted a retrospective observational cohort study based on health insurance claims data. After we identified patients with depression who were on sick leave due to a mental disorder in 2015, we applied entropy balancing to adjust for selection effects and employed chi-squared tests to compare guideline adherence of the received treatment between PNP, the GP program and UC. Subsequently, we applied an extended cox regression to assess need-orientation by comparing the relationship between accumulated sick leave days and waiting times for psychotherapy across health plans.

**Results:**

*N* = 23,245 patients were included. Regarding guideline adherence, we found no significant differences for most severity subgroups; except that patients with a first moderate depressive episode received antidepressants or psychotherapy more often in UC. Regarding need-orientation, we observed that the effect of each additional month of sick leave on the likelihood of starting psychotherapy was increased by 6% in PNP compared to UC.

Irrespective of the health plan, we found that within the first 12 months only between 24.3 and 39.7% (depending on depression severity) received at least 10 psychotherapy sessions or adequate pharmacotherapy.

**Conclusions:**

The PNP contract strengthens the relationship between sick leave days and the delay until the beginning of psychotherapy, which suggests improvements in terms of need-oriented access to care. However, we found no indication for increased guideline adherence and – independent of the health plan – a gap in sufficient utilization of adequate treatment options.

**Supplementary Information:**

The online version contains supplementary material available at 10.1186/s12888-020-02995-1.

## Background

Major depressive disorder is a highly prevalent disorder with a lifetime prevalence of 14.6% in high income countries [1] and a 12-month prevalence of 7% in Germany [2]. It is associated with a reduced quality of life [3], cognitive impairment [4], high treatment costs and losses in productivity [5–8]. Moreover, its impact on the global disease burden increased rapidly over the past decades [9, 10]. Worldwide projections indicate that unipolar major depression will be the leading cause of disease burden by 2030 [11]. Consequently, health care systems need to identify options to provide more efficient treatment.

A promising approach to improve depression treatment are integrated and collaborative forms of care [12–16]. In Germany, however, mental health care funding and organization of services does not effectively incentivize the collaboration between outpatient care providers and intersectorial collaboration with hospitals. Although there is a clear trend towards ambulatory health care centers, 49% of all outpatient physicians and psychotherapists still autonomously manage their own medical practice [17]. In addition, hospitals are funded separately and are staffed by different teams [18]. Hence, there are few natural ways to collaborate.

Germany’s mental health care system lacks an effective mechanism to distribute the available resources according to symptom severity. As a result, in particular patients with severe depression are often treated insufficiently [19–21]. Furthermore, the duration of disease burden is often prolonged by long waiting times for psychotherapy [22–25].

To address these issues, the AOK Baden-Württemberg (BW), the largest statutory health insurance (SHI) company in southwest Germany, developed and implemented the integrated care psychiatry-neurology-psychotherapy (PNP) contract. The PNP contract aims to facilitate the cooperation between general practitioners (GPs), mental health specialists and the psychosocial service of the AOK BW. The latter assists during stressful periods in life and arranges specialist appointments if necessary.

Furthermore, the PNP contract promotes guideline adherence and strives for a more needs-oriented allocation of resources and reduced waiting times for patients with acute or severe mental disorders. It is one of several selective contracts, which are offered collectively to insurees of the AOK BW in the specialists’ program as an alternative to usual care (UC). Other selective contracts within the specialists’ program exist for e.g. gastroenterology, cardiology and orthopedics. On the provider’s side, these selective contracts change regulations for the corresponding group of specialists in the outpatient sector if they decide to enroll. Structurally, patient participation in the specialists’ program requires enrollment in the GP program, in which patients accept gatekeeping i.e. - in contrast to UC - patients need a referral from their GP to gain access to specialist care.

The utility of gatekeeping is still debated [26–29]. For mental health care, gatekeeping might discourage some patients from seeking professional help, which would be hazardous given that treatment rates for patients with mental disorders are already low [30–32]. However, depending on how gatekeeping is implemented, it might also place GPs at the center of the outpatient network and emphasize their role in coordination and resource allocation, which might result in a more responsible use of specialist resources.

The GPs ability to provide optimal care and coordinate treatment for patients with chronic disorders is to some extent reliant on sufficient interactive communication and collaboration with specialists [33, 34]. Moreover, patients could be encouraged to participate in a program that requires the additional step of going to a GP first, if specialists favor referred over non-referred patients when scheduling patient appointments. Consequently, the specialists’ program might be an important extension to the GP program, because participating mental health specialists commit to additional guidelines on accessibility for referred patients and share additional information on those patients with the GP.

So far, a provider survey [35] and a cost comparison [36] were published from the multiperspective evaluation of the PNP contract [37], but both studies have not obtained reliable evidence for potential mediators of the contract. In the survey, providers reported general satisfaction with the contract and the vast majority stated that the treatment of patients with chronic conditions or an acute crisis had improved e.g. due to need-oriented and timely access to care, but since providers financially benefit from the contract they might be biased. The cost comparison found that the PNP contract reduced producitivty losses (e.g. sick leave days due to common mental disorders) when compared to UC or the GP program, but the study was solely outcome-oriented. Hence, in this paper, we aim to identify relevant mediators of the contract. For this purpose, we focus on patients with depression – the largest homogenous subgroup of our trial sample – and assess two potential mediators that could explain the results of the cost comparison. Primarily, we want to answer the following research questions regarding the mediators guideline adherence and need-oriented access to psychotherapy (i.e. whether waiting times depend on depression severity and relative urgency):

1. Does the PNP contract in the specialists’ program increase the rate of patients with depression treated according to guidelines compared to UC and the GP program?
2. Does the PNP contract in the specialists’ program better achieve need-oriented access to psychotherapy for patients with severe or acute episodes of depression compared to UC and the GP program?

## Methods

### Study design and sample

We conducted a retrospective observational cohort study based on SHI claims data from 2014 to 2016 of the AOK BW to identify, balance and compare three cohorts of either UC, GP or specialists’ program patients with depression. In view of our aim to identify mediators that explain our previous findings, we wanted to maintain comparability to our previous analysis by using similar inclusion criteria [36]. Consistently, we only included patients that received a sick leave diagnosis in 2015 due to one of the mental disorders listed in Table 1. These mental disorders were selected, because they often co-occur with one another, are often treated by the same specialists and are important from a payer’s perspective due to high costs or prevalence.

**Table 1:** Inclusion criteria

1. Age ≥ 18 years	
2. Continously insured by the AOK BW	
3. Place of residency in Baden-Württemberg (southwestern Germany)	
4. Sick leave diagnosis in 2015 due to alcohol abuse (F10.x), schizophrenia (F20.x), bipolar disorder (F31.x), depressive episode (F32.x), recurrent depressive disorder (F33.x), dysthymia (F34.1), phobic anxiety disorder (F40.x), other anxiety disorders (F41.x), adjustment disorder (F43.2), or somatoform disorder (F45.x) and no sick leave diagnosis in the previous 12 month because of a mental disorder (Fxx.x)	
5. Inpatient depression diagnosis or at least two outpatient depression diagnoses (F32 or F33) within the quarter in 2015, in which the patient received the sick leave diagnosis, or the two subsequent quarters	
6. Complete cost data, plausible length of stay and cost values (e.g. aggregated cost data had to be positive)	

AOK is a large statutory health insurance company in Germany, BW Baden-Württemberg (region in southwestern Germany)

Sick leave diagnoses in Germany are documented with a fixed date, which we interpreted as the onset of a new episode of a mental disorder. This approach allowed us to assess waiting times as the difference in days between that sick leave diagnosis and the first therapy session. We excluded patients if they received a sick leave diagnosis due to any mental disorder during the 12-month-preperiod, in order to only include patients with an acute episode.

We classified all individuals according to the program enrolled in the quarterly period of their index sick leave date as either UC, GP or specialists’ program patients. The individual preperiod was used to determine patient characteristics and assess each individual’s health status. Subsequently, we balanced the groups with regard to those characteristics via Entropy Balancing (EB) [38], in order to alleviate the selection bias.

In view of our focus on patients with depression and concerns regarding the specificity of a single claims data diagnosis [39, 40], we additionally checked whether patients received either an inpatient or at least two outpatient depression diagnoses in two distinct quarters. These diagnoses had to be documented within the sick leave quarter or the two subsequent quarters.

For the first research question on guideline-adherence, we determined depression severity to examine severity-dependend guideline recommendations [41]. We decided to categorize individuals based on the most severe diagnosis they received (assuming recurrent > first episode and severe > moderate > mild). Patients with mild vs. moderate vs. severe episodes were separately grouped depending on whether it was their first or a recurrent episode, which resulted in six subgroups. All groups were compared cross-sectionally across health plans in different indicators for guideline adherence (e.g. adequate pharmacotherapy or psychotherapy rates).

For the second research question on timely and need-oriented access to psychotherapy, we focused on patients with moderate and severe depression, because for these patients psychotherapy should always be considered and offered. According to the National Clinical Practice Guideline [42], all moderate cases should be offered either psychotherapy or pharmacotherapy and all severe cases should be offered a combination of both, while patients with mild depression can also be monitored or supported through other means. We assessed this second question in longitudinal models, because waiting times and our proxy for need were time-dependent. Since longitudinal models are capable of incorporating time-varying predictors, we allowed for quarterly changes in the health plan status. This means that the composition of the three health plan groups might change each quarter. Hence, we applied EB for each individual quarter, so the groups remain comparable despite the fact that patients switch between health plans.

### Interventions

Table 2 provides a brief overview of the three programs. A more comprehensive comparison can be found in the study protocol [37]. The GP program aimed to implement gatekeeping and mandatory quality circles on drug therapy, which might influence resource allocation and the provision of pharmacotherapy. These changes also affect patients in the specialists’ program, because they are simultaneously enrolled in the GP program.

**Table 2:** Summary of the differences between the health care contracts

	Usual care	General practitioner program	Specialists’ program
Gatekeeping	none (i.e. free choice between all health care providers that are licensed to bill their services to a SHI)	commitment to first seek help from one particular GP enrolled in the program (exception: e.g. emergencies, gynecologist) free choice between specialists if patients are referred by the GP	commitment to first seek help from one particular GP enrolled in the program (exception: e.g. emergencies, gynecologist) free choice between participating specialists if patients are referred by the GP
Differences in the organization and payment of psychotherapy	The first 5 preparatory sessions are compensated to a lesser extent (64 €) when compared to regular sessions (84 €) • review process for approval of long-term psychotherapy • more highly frequented therapy possible for people with severe mental disorders (max. 80 h for behavioral therapy, max. 100 for psychodynamic therapy and max. 300 for analytic therapy) • waiting time until initial assessment on average approx.. 11,4 weeks in BW [43]	The first 5 preparatory sessions are compensated to a lesser extent (64 €) when compared to regular sessions (84 €) review process for approval of long-term psychotherapy more highly frequented therapy possible for people with severe mental disorders (max. 80 h for behavioral therapy, max. 100 for psychodynamic therapy and max. 300 for analytic therapy) waiting time until initial assessment on average approx.. 11,4 weeks in BW [43]	stepped payment scheme (105 € for urgent cases for max 10 sessions, 90€ for the following max 20 sessions, 84.5€ for the following sessions) higher payment for group psychotherapy after 60 h of therapy only therapy with a low frequency (max. 6 times per quarter) is billable (exception: personality disorders or if the diagnosis changes), application for an extension (e.g. to prevent relapse) is possible start of psychotherapy within 4 weeks after established diagnosis, for acute cases: initial session within 3 days; start of psychotherapy within 7 days after established diagnosis
Differences in the organization and payment of psychiatry			**Psychiatry:** additional supplement (e.g. for counseling, prescription of discounted medication), limit of waiting time to 30 min, for acute cases: first appointment within the same day
Differences in the organization and payment of neurology			**Neurology:** additional supplements (e.g. Multiple Sclerosis counselling: 6 units of 20 min per year, provision of assistants), limit of waiting time up to 30 min, for acute cases: first doctor’s appointment within the same day

AOK is a large statutory health insurance company in Germany, BW Baden-Württemberg (region in southwest Germany), GP general practitioner, PNP psychiatrist neurologist psychotherapist contract, All actual € values refer to the costs in the first Quarter of 2015

The PNP contract implemented new legal regulations on accessibility in neurology and psychiatry and readjusted multiple regulations in the field of psychotherapy when compared to UC or the GP program. Specifically, the PNP contract incentivizes group psychotherapy, allows additional treatment options and drops the time-consuming review process for the approval of psychotherapy. Moreover, it implements a stepped payment scheme that grants a substantial surplus for the first 10 psychotherapy session for patients suffering from acute disorders (e.g. moderate and severe depression), but only if these are provided within a limited time period. Together, these regulations should ensure faster treatment for undersupplied patient groups.

### Group formation and balancing of covariates

The samples in the three health plans were systematically different with regard to various characteristics at baseline – irrespective of whether we analyzed the six subgroups or all moderate and severe cases. Thus, in all seven samples, we applied EB [38] to reweight the control groups (UC and GP), so that the mean, variance and skewness of potentially confounding covariates for the 365 day preperiod were almost identical across health plans.

In the longitudinal models, we allowed for quarterly changes in the health plan status to incorporate switching between health plans, which means that the composition of the groups (UC, GP and specialists’ program) might vary on a quarterly basis. We considered the health plan status at the index date, after 91 days, 182 days and 273 days, and reweighted the groups separately in each of the resulting quarters of the observational period.

We considered various potential confounders. First, we balanced for healthcare costs and service use, which includes sick pay, outpatient, inpatient and medication costs, sick leave days, hospital days, antidepressant prescriptions within the last 6 months and psychotherapy within the last 2 months. Second, we balanced the severity of depression and the source of the diagnosis (i.e. inpatient vs. outpatient). Third, we balanced comorbidities, which includes anxiety disorders, somatoform disorders and all elixhauser subscales based on ICD-10-codes [44] documented in the inpatient or outpatient sector. However, if less than 40 patients were affected from a certain comorbidity (e.g. HIV is extremely rare), we did not balance for it, because EB might assign high weights to individual patients in an attempt to balance rare disorders, which would make the analysis prone to outliers. Lastly, we balanced for age, sex, urbanization of the region of residence [45] and the quarter of the index date.

### Outcomes of interest

First, we test for increased adherence to guidelines. To that end, we compare the proportion of patients receiving pharmacotherapy, psychotherapy or a combination of both in the six subgroups, because these rates indicate over-, under- or misuse of resources related to depression severity [41]. Moreover, we compare treatment intensity by assessing the number of psychotherapy sessions and the rates of adequate pharmacotherapy. We defined sufficient pharmacotherapy as over 180 daily defined dosis (DDD) [46] of antidepressants. We chose this threshold, because it represents the minimum of what is recommended by guidelines – i.e. about 4–6 weeks of acute therapy and 4,5–5 months of maintenance therapy [41]. Moreover, the 12-month observation period was to short to test for a more conservative threshold. However, this definition would not account for inpatient prescriptions (not coded in German claims data) and risky prescription patterns such as simultaneous treatment with two antidepressants. Hence, we developed a complex algorithm based on the National Clinical Practice Guideline [42] (see supplemental Table 1) to handle these cases adequately.

Second, we test whether the PNP contract facilitates need-oriented access to psychotherapy. Need-oriented access implies that the burden of the patient should have a considerable influence on waiting times. Since the burden cannot be measured directly, we used sick leave days due to common mental disorders (i.e. F10, F20, F31, F32, F33, F341, F40, F41, F432, F45) as a proxy. Sick leave days cumulatively add up over the observation period and we accounted for each individual 5 day change in sick leave days in our longitudinal model. Then, we examined whether the effect of these accumulated sick leave days on waiting time for psychotherapy and on the frequency or sessions was stronger for patients in the specialists’ program compared to UC or the GP program.

Moreover, we compared the amount of sessions. We considered all services that take at least 50 min and double-counted group sessions that took at least 100 min (we coded a delay of 0.01 days for the second session), and extracted the exact date of each session, in order to examine the waiting time for the initial session and the delay between subsequent sessions.

### Statistical approach

For the analysis of increased guideline adherence, we conducted chi-square tests with the Rao-Scott correction to compare treatment rates and the proportion of sufficiently treated patients between health plans [47, 48]. For the number of psychotherapy sessions, we used two-part models, in order to simultaneously estimate the probability of patients initiating psychotherapy (binomial distributed), and if so, model the number of sessions (truncated, negative binomial distributed).

For the analysis of need-oriented access to psychotherapy, we applied the Prentice, Williams and Peterson (PWP) gap time model with robust standard errors [49] – an extension of the Cox-proportional hazard model. The gap time was defined as the delay in days from the previous sessions or the delay from the index sick leave date for the initial session.

## Results

### Sample

*N* = 23,245 patients met the inclusion criteria (see Table 1). The study cohort had a mean age of 45.48 years (SD 11.76) and 38.46% were male. Regarding the cases with a first episode of depression, 5918 (25%) were categorized as moderate and 2458 (11%) as severe cases. Regarding the cases with a recurrent episode, 4879 (21%) were categorized as moderate and 3696 (16%) as severe cases. Patients with mild depression were not analyzed separately, because the sample size (GP: *n* = 321, specialist’ program: *n* = 172 and UC: *n* = 443) was insufficent to balance the selected covariates. Thus, we had to group all mild (*n* = 936) and non-specified cases (*n* = 5358) to achieve convergence. Moreover, for patients with a recurrent moderate, mild or non-specified and a first severe episode, convergence of EB for the subgroup in the GP program required dropping the skewness requirement, which means that these subgroups are only comparable to the PNP sample with regard to the mean and variance of the included covariates. We do not discuss the differences between health plans for the residual subgroup of mild and non-specified cases, because no clear evidence-based recommendations on treatment strategies exist for non-specified cases. Instead, we provide the results on the these cases in the supplement (see supplemental figure 1 and supplemental Tables 3, 4, 5, 6).

For the second question, we assessed all moderate and severe cases. This sample had a similar average age of 45.58 years (SD = 11.70). About 38.6% were male, resulting in a female to male ratio of 1.59. The supplemental Table 2a and b provide all unadjusted descriptive statistics for the 12-month preperiod on the three health plan groups. These statistics indicate that the group in the specialists’ program had a higher utilization of health services and a worse health status at baseline. However, these differences disappeared after the EB adjustment.

### Differences in guideline-adherence

**Fig. 1:**
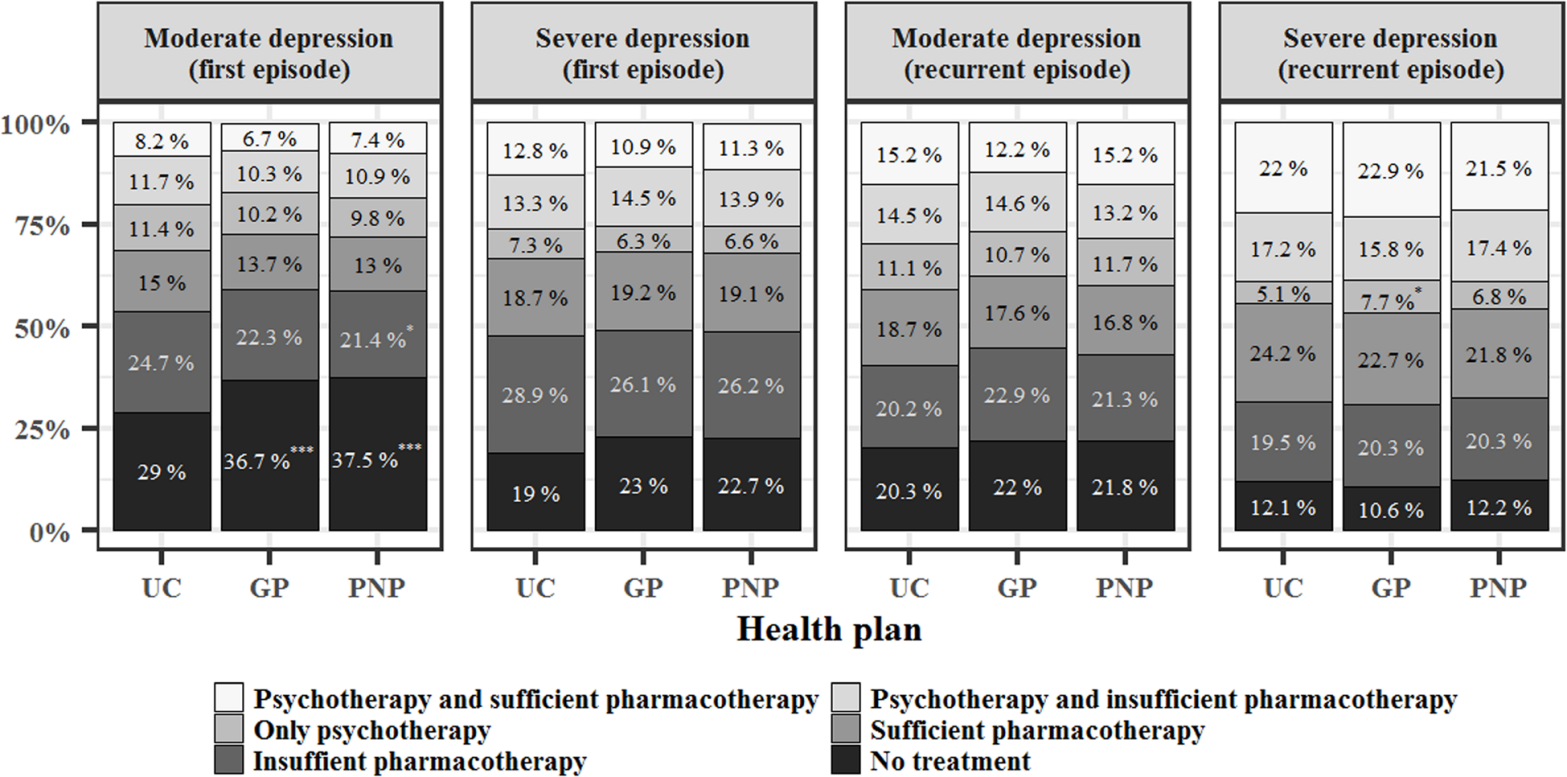
Stacked barplot comparing treatment type by severity and health plan. *GP* general practitioner program, *PNP* psychiatry-neurology-psychotherapy contract, *UC* usual care. Psychotherapy was defined as at least one session and phamacotherapy as at least one antidepressant prescription within the 12-month observational period. We calculated the chi-square test with the Rao-Scott second order correction, in order to compare the proportion of the six different treatment types in the three health plans. *N* = 5.918 had a first episode and *N* = 4.879 a recurrent episode with a moderate severity. *N* = 2.458 had a first episode and *N* = 3.696 a recurrent episode with severe depression, *p < .05, **p < .01, ***p < .001. For patients that received multiple diagnoses from different sources, we assigned the source with the highest validity (assuming psychiatrists/psychotherapists > neurologist > primary care physician > other discipline)

In the following figures, patients within the specialists’ program are referenced as the PNP group, because the PNP contract regulates mental health care for these patients. We found mostly negligible differences between the three health plans in treatment rates (see Fig. 1). After applying a Bonferroni-correction to the significance level, which adjusted α for the 48 different comparisons in our assessment of treatment type (α approx. 0.001), we found that only the difference in the proportion of untreated patients with a first moderate episode remained significant. We found that in UC only 29% were not treated with either an antidepressant or psychotherapy, whereas this rate was 7.7% higher in the GP program, F (1,4681) = 19.155, *p* < 0.001, and 8.5% higher in the PNP group, F (1,4016) = 22.988, *p* < 0.001.

**Fig. 2:**
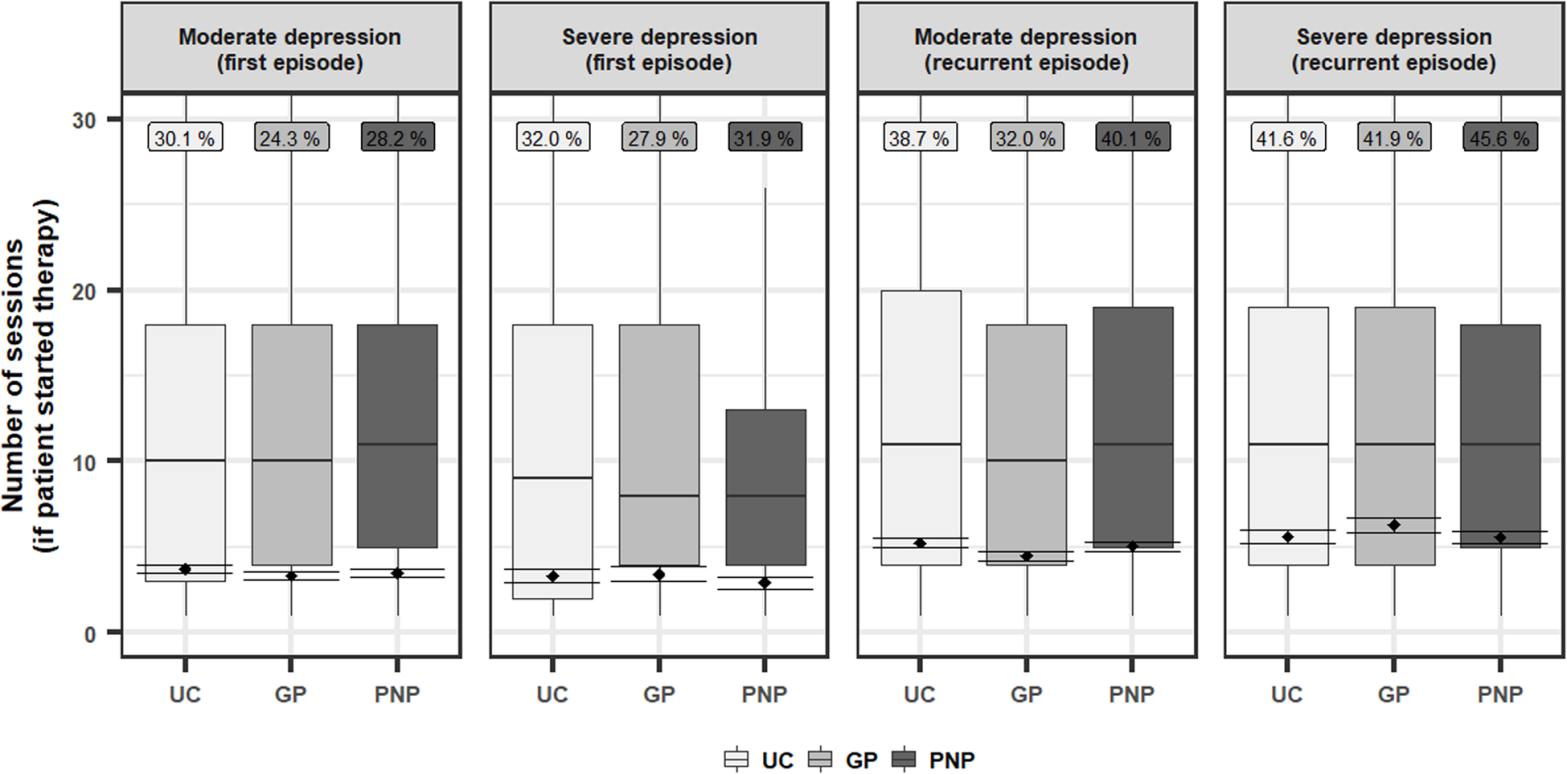
Boxplots on the amount of therapy sessions by severity and health plan. Only shows patients with at least 1 therapy session within the observational period. The smaller boxes above the boxplots present the proportion of patients that receive psychotherapy. A session was defined as a service that requires 50 min interaction with a licensed psychotherapists. We plotted the estimated marginal means and confidence bands (± 1 Standarderror) from the two-part model. GP general practitioner program, PNP psychiatry-neurology-psychotherapy contract, UC usual care. *N* = 5.918 had a first episode and *N* = 4.879 a recurrent episode with a moderate severity. *N* = 2458 had a first episode and *N* = 3.696 a recurrent episode with severe depression. For patients that received multiple diagnoses with different severities, we assigned the diagnosis with the highest severity (assuming F33 > F32 and severe > moderate > light)

Figure 2 compares the distributions of the number of received therapy session between different severities and health plans. We added estimates from the two-part models. There were no significant differences in the marginal means (see supplemental Table 3), the probability to receive at least one therapy session or the amount of sessions conditional on patients receiving at least one therapy session. The model coefficients of the appertaining two-part model are provided in supplemental Table 4.

### Differences in need-oriented access to psychotherapy

**Fig. 3:**
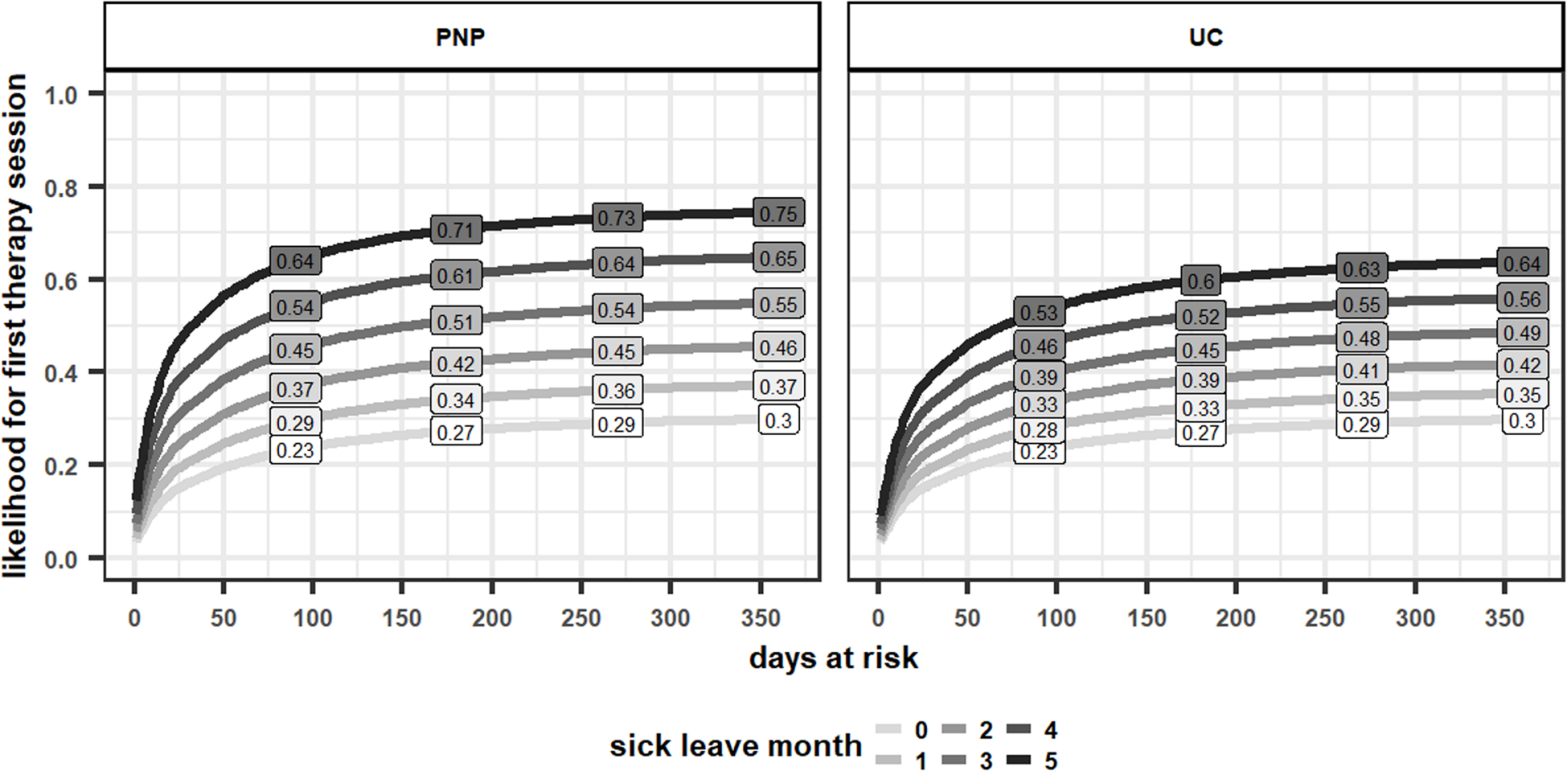
Reverse survival curves for the first therapy session. PNP psychiatry-neurology-psychotherapy, UC usual care. We predicted the likelihood for patients that were on sick leave 1, 2, 3, 4 or 5 month for each health plan based on the coefficients of the PWP-gap time model. The labels show the estimated likelihood of receiving the first psychotherapy session within 90, 180, 270 and 360 days. The curves were plotted only for the comparison of usual care (UC) with the specialists’ program

Table 3 shows the results of the PWP-gap time model. In the specialists’ program sick leave months have a stronger effect on the hazard of the first psychotherapy session than in UC, β = .06, z = 4.02, *p* < .001. Specifically, patients in the specialists’ program have an expected 6% increase in the hazard of receiving the first session for each additional month on sick leave when compared to UC. This effect is illustrated through reverse survival curves in Fig. 3.

**Table 3:** Results of the PWP-gap time model

Outcome	First session		All sessions	
	Exp(β) (rse)	Sig.	Exp(β) (rse)	Sig.
Sick leave month	1.23 (0.01)	***	1.23 (0.01)	***
PNP	1.00 (0.04)		1.00 (0.04)	
GP	0.93 (0.05)		0.93 (0.05)	
Sick leave month*PNP	1.06 (0.01)	***	1.06 (0.01)	***
Sick leave month *GP	1.01 (0.02)		1.01 (0.02)	
PNP*Therapy session (2–6)			1.02 (0.05)	
PNP*Therapy session (over 6)			0.96 (0.06)	
Sick leave month*Therapy session (2–6)			0.81 (0.01)	***
Sick leave month*Therapy session (over 6)			0.83 (0.01)	***
GP*Therapy session (2–6)			1.10 (0.06)	
GP*Therapy session (over 6)			1.07 (0.07)	
Sick leave month*PNP*Therapy session (2–6)			0.96 (0.02)	*
Sick leave month*PNP*Therapy session (over 6)			0.94 (0.02)	***
Sick leave month*GP*Therapy session (2–6)			1.01 (0.02)	
Sick leave month*GP*Therapy session (over 6)			0.99 (0.02)	

*GP* General practitioner program, *PNP* Psychiatry-neurology-psychotherapy contract, *PWP* Prentice, Williams and Peterson, rse robust standard error, *UC* Usual care, one sick leave month equals 30 sick leave days, *N* = 16,951, *p < .05, **p < .01, ***p < .001

Conversly, we found a counteracting effect for the subsequent sessions. Thus, the initially higher effect for the specialists’ program is reduced in sessions 2 to 6, β = −.04, z = − 2.26, *p* = .0239 and in the following sessions, β = −.07, z = − 3.95, *p* < .001. For the GP program, we found no signficant differences in the waiting time or the frequency of sessions. We provide additional reverse survival curves that illustrate the effect for the subsequent sessions in Figs. 2 and 3 of the supplement.

### Post-hoc analysis

Regarding the lower treatment rates in the GP and the specialists’ program for patients included due to a first episode of moderate depression, we examined whether single prescriptions and psychotherapy sessions or comprehensive services were prevented. Thus, we compared the likelihood of receiving at least 10 psychotherapy sessions, adequate pharmacotherapy or both (see supplement Table 6). Testing for these stricter outcomes, we no longer found significant differences in the rates of untreated patients between the specialists’ program and UC.

Moreover, we tested for negative effects of these reduced treatment rates on productivity losses or hospitalization. For that purpose, we applied a censored, negative binomial model to compare the sick leave days in the 12-month observational period in the four subgroups with either moderate or severe depression and compared hospitalization rates via chi-square test. For patients with a first episode of moderate depression – for whom we observed lower treatment rates – sick leave days were 37.68 days lower in the specialists’ program than in UC, t (5918) = − 6.21, *p* < .001, and 14.08 days lower than in the GP program, t (5918) = − 2.69, *p* = .0071 and hospitalization rates were descriptively lower. Consequently, we found no evidence that these lower treatment rates have a negative effect on productivity losses or hospitalization.

## Discussion

### Interpretation of the results

In this analysis, we aimed to find mediators that could explain the reduction of sick pay and sick leave days due to common mental disorders in the specialist program, which we found when we compared the costs for the treatment of common mental disorders between the specialist program, the GP program and UC [36]. We examined whether the specialists’ program increased the rate of patients that received guideline adherent depression treatment or if the regulations in the specialists’ program facilitate timely and need-oriented access to psychotherapy. Increasing the rates of patients, who receive guideline adherent treatment, would be a relevant improvement in consideration of the low rates of appropriately treated patients with mental disorders in UC [32, 50]. Moreover, preferred access or reduced waiting times for psychotherapy for patients with severe and acute depression could be advantageous, because in UC patients with high symptom severity are insufficiently treated [19–21] and patients with high severity might benefit to a larger extent from psychosocial interventions [51, 52].

Regarding the guideline adherent treatment rates, we found that the likelihood of receiving psychotherapy, pharmacotherapy or both was similar across health plans for most severity subgroups. Accordingly, we found no differences in the amount of sessions or the appropriateness of pharmacotherapy. However, in the GP and the specialists’ program fewer patients with a first moderate episode received guideline recommended treatment. In view of the low treatment rates for patients with mental disorders [50, 53], a further decline in treatment rates could be a cause for concern, but our post-hoc analysis showed that the respective subgroup did not differ significantly across health plans when assessing more strict criteria – i.e. adequate pharmacotherapy or above 10 psychotherapy sessions. Moreover, this subgroup developed comparatively well in in terms of sick leave days and was hospitalized less often in the specialists’ program. Consequently, we assume that the decline in treatment rates could be attributable to the reduced utilization of specialist care observed in the GP program [54]. Lower utilization rates for specialists care are intended and expected in a gatekeeping framework [26] and as assumed by stepped care interventions [55] they do not have to result in lower remission rates if they only impact minor interventions that the GP can provide as well. Nevertheless, it might be worth exploring in future analyses and reforms of the PNP contract, whether a larger proportion of patients in this subgroup should be referred to specialists or receive psychoeducation and information on potential treatment options.

Regarding timely and need-oriented access to psychotherapy, we demonstrated that sick leave months had a significantly stronger effect on the likelihood of starting psychotherapy in the specialists’ program when compared to UC. Each additional sick leave month due to mental disorders increased the likelihood for starting psychotherapy by 23% in UC, but by more than 30% in the specialists’ program. As a result, waiting times for acute and burdened cases were lower in the specialists’ program. For example, the PWP model would predict that a patient who has been on sick leave at least 90 days in the follow-up period (32.4% of our sample) has a probability of 39% in UC and of 45% in the specialists’ program to receive an initial session within the next 90 days. While this effect appears small, we point out that we overestimate the actual waiting time in all groups, because we could only choose the initial sick leave diagnosis and not the moment that patients intended to start psychotherapy as the beginning of the waiting period. Hence, the relative benefit of PNP in terms of waiting times for psychotherapy should be higher – especially considering that patients with more sick leave days are common and benefit even more (see supplemental figure 4) and treatment rates have an upper constraint due to patients actively deciding against treatment. Reducing waiting times for patients who are contributing the most to high productivity losses could explain the reduction of sick leave days and sick pay observed in our recent analysis [36]. Moreover, this result is plausible considering that the specialist program changed access guidelines with regard to psychotherapy and implemented a stepped payment scheme that incentivizes timely treatment for acute and severe cases [37] Moreover, in a recent provider survey enrolled psychotherapists reported that the PNP contract facilitates timely and need-oriented access [35].

On account of the PWP-model’s ability to handle repeated events, we also examined the delays between subsequent therapy sessions. We found that the relative benefit of the specialists’ program declines in these sessions. Both, the hazard increasing ratio of 1.06 (initial session) and the hazard decreasing ratio of 0.96 (2–6 session) or 0.94 (above 6 sessions) constitute the effect of sick leave month for subsequent sessions in the specialists’ program. These opposing forces almost cancel each other out. Thus, the differences between health plans in the effect of sick leave month on the subsequent sessions appears to be less relevant than the effect on the initial session (see Figs. 2 and 3 of the supplement).

### Implications beyond the evaluation of the alternative health plans

First, our findings indicate that it is possible to categorize patients with depression by severity based on claims data. More severe forms of depressions were associated with greater treatment intensity, more comprehensive services and on average more sick leave days. Nevertheless, sick leave days still vary substantially within subgroups and there is large overlap between the distributions of sick leave days by severity (see supplemental figure 4), which suggests that accumulated sick leave days are a more precise proxy for subjective burden. Nonetheless, the validity of documented claims data diagnoses of mental disorders still needs to be confirmed and compared with assessments based on primary data – especially when documented by GPs [56].

Second, outpatient treatment rates remain low. At a reasonable threshold for a sufficient treatment within the first 12 months (at least 10 sessions or adequate pharmacotherapy), we found that only 24.3% with a first moderate and 27.6% with a first severe episode received sufficient treatment, whereas 32.5% with a moderate and 39.7% with a severe recurrent episode received adequate treatment (see supplement Table 6). Although these results are somewhat misleading for patients with recurrent severe depression, because they are often treated in hospitals (see supplemental Table 5), they are worrying for the other severity subgroups, because the distributions of sick leave days indicate that these subgroups experience high subjective burden as well. Furthermore, we found substantial differences in the proportion of patients who initially started pharmacotherapy and the proportion of patients who receive sufficient antidepressants for at least 180 days of treatment, which could indicate – in accordance with studies on chart reviews or interviews [57, 58] – that many patients discontinue their antidepressant treatment. In addition, the treatment intensity required for remission is presumably higher and our sample is only a subset of those suffering from depression, because many do not seek professional help [30–32]. It is unclear, whether this issue can be solved solely by implementing alternative health plans, because several studies indicate that the attitude towards mental disorders [59–61] and a lack of knowledge about mental health problems [62] contribute to insufficient or delayed utilization of mental health care. These attitudinal barriers are probably difficult to overcome simply by changing regulations.

Third, although the mentioned problems may only partially depend on the provision of care, our results indicate that shorter waiting times and a more needs-oriented allocation mechanism could improve the situation. This is supported by the result that patients with a first moderate episode appear to benefit from the specialists’ program, presumably because more acute cases profit from shorter waiting times.

### Strenghts and limitations

Our approach of using claims data has some advantages over the use of primary data. First, there is no bias due to drop out or recruitment. Second, claims data offer valuable information on treatment intensity (e.g. prescribed DDD of a particular agent, received therapy sessions) with remarkable accuracy and in the context of depression treatment several objective process and quality indicators can be measured, which are difficult to obtain or easily distorted through recall biases when questionaires are used. Third, we could investigate the order of prescribed medications and the delay between psychotherapy sessions, which allowed us to detect inadequate prescription patterns and assess waiting times and treatment frequency. Moreover, we could account for switching between contracts and the accumulation of sick leave days in the PWP-model. Nonetheless, our study has its limitations. First, we could not observe the completion of psychotherapy for the entire sample within 12 months, because the acute phase of psychotherapy takes about 1.5–3 months and the maintenance phase about 8–12 months [42]. Moreover, our proxy for adequate pharmacotherapy required 180 DDD of a single agent, which means that patients that legitimately switch between medications due to adverse effects or patients that start their treatment 8–9 months after their sick leave date, might be classified as insufficiently treated, although they will receive sufficient treatment in the subsequent months. Furthermore, treatment is often delayed by waiting times and the thorough cognitive process that precedes the decision for or against pharmacotherapy or psychotherapy [63]. Thus, a longer follow-up period may be necessary, if an accurate estimation of adequately treated patients is of interest.

Second, claims data are mainly used for accounting purposes and lack clinical information. Therefore, it can be difficult to define quality criteria, because the adequacy of a patients’ treatment depends on the individual history (e.g. prior experience with antidepressants or psychotherapy) and on patient characteristics (e.g. onset, severity and course), which are only partially observable in claims data. Hence, clearly defined cut-off points such as 180 DDD of antidepressants are imperfect proxies for appropriate treatment, which can only detect larger differences in guideline adherence or treatment quality. Furthermore, we were not able to assess adherence beyond patients picking up their prescriptions and appearing to psychotherapy sessions. In view of the large difference between the rate of sufficiently treated patients and the rate who initially started an appropriate form of treatment, it would have been useful to collect primary data to understand treatment delays and why patients switched to another agent or even discontinued their treatment. Notably, we can not dismiss the possibility that some patients should be treated differently according to guidelines, because we included patients with psychiatric comorbidities and patients that originally received a sick leave diagnosis for a psychiatric disorder other than depression. For these patients, we can not differentiate between comorbidity and primary disorder, because the chronology of the documented diagnoses is not always meaningful in claims data. We decided to control for these comorbidities rather than to exclude patients with these comorbidities, because a) comorbidities are common in depressive disorders (i.e. excluding these patients could reduce the generalizability) and b) our main interest was to compare health plans (therefore, our priority was to generate comparable groups of a sufficient size to enable balancing and severity specfic assessments). Nevertheless, we point out that some disorders affect guideline recommendations. For instance, if substance abuse develops in the course of a depressive disorder in an attempt to self-medicate, qualified withdrawal, rehabilitation and long-term monitoring should be prefered over the type of treatment we assessed [42, 64]. Similar subtleties apply to anxiety disorders or in particular to severe mental disorders (see supplemental Table 1a on comorbidity rates for all moderate and severe cases in our sample).

Third, documented diagnosis may be influenced by financial incentives or legal regulations [65]. For instance, non-specified depression diagnoses are no longer sufficient to bill psychotherapy sessions in the PNP contract, which might affect the coding behavior of GPs for patients referred to psychotherapists, although the GP’s compensation does not directly depend on it.

Fourth, some potential mediators such as the collaboration between providers are difficult to measure based on claims data. We do not know, whether the quality of reports, the exchange between GPs and mental health specialists or the collaboration with care extenders improved, although this aspect is addressed to some extent in a complementary patient and provider survey [37].

Fifth, there were notable differences between the groups at baseline. Similar to our previous analysis [36], we again found that patients in the specialists’ program had on average a worse health status e.g. higher utilization, more recurrent episodes and more somatic comorbidities (see supplemental Table 1a). This selection bias is expectable, since the insured can choose freely between the different health plans. However, EB or another matching technique is necessary to adequately controll for this bias, which is difficult if there are large difference at baseline or if the sample size is insufficent to find enough comparable cases. In this study, we could not sufficiently balance the skewness of the control variables for patients with a recurrent moderate, mild or non-specified and a first severe episode effect of the GP program. Consequently, the results for these subgroups should be interpreted with caution.

Lastly, we observed the effects in a competitive market. Thus, the advantage of the specialists’ program may depend on the financial benefit of accepting PNP patients over other patients.

## Conclusion

Our paper showed that the new regulations in the PNP contract reduce waiting times for psychotherapy for high-need patients with depression, which could be a substantial improvement from a patient perspective, because long waiting times are one of the main structural barriers in mental health care. Particularly, patients with a first moderate episode appear to improve considerably in terms of sick leave. However, we found no evidence that the PNP contract improves guideline adherence or the outcomes for chronic and severe cases and – independent of the health plan – our results suggest a gap in sufficient utilization of adequate treatment options. We recommend conducting longterm studies that merge primary with secondary data. These are needed to observe the completion of psychotherapy, to assess the validity of diagnostic information from secondary data sources and to learn why a large proportion of patients does not receive a guideline adherent form of depression treatment.

## Supplementary Information

**Additional file 1: Table 1**a: Unadjusted statistics for the 12-month period before the index diagnosis. Table 1b: Unadjusted statistics for the 12-month period before the index diagnosis. **Figure 1**. Plots on treatment type, the number of therapy sessions and sick leave days for patients with mild or non-specified depression. **Table 2**. Estimated marginal means for sick leave days and therapy sessions in the 12-month after the index diagnosis. **Table 3**. Model coefficients for the number of therapy sessions in the 12-month after the index diagnosis. **Figure 2**. Reverse survival curves on the effect of sick leave month on the likelihood of receiving another therapy session in PNP compared to the effect in UC for the second to fifth session. **Figure 3**. Reverse survival curves on the effect of sick leave month on the likelihood of receiving another therapy session in PNP compared to the effect in UC for the sixth and the subsequent sessions**. Figure 4**. Boxplots on the number of sick leave days. **Table 4**. Model coefficients for sick leave days in the 12-month after the index diagnosis. **Table 5**. Weighted proportion of adequately or inadequately treated patients in the outpatient sector and the hospitalization rate compared between health plans in various subgroups. **Table 6**. Weighted proportion of adequately or inadequately treated patients by severity subgroups.

## Data Availability

Data are owned by the German statutory health insurance AOK BW. To request the data please contact the institutional body of the AOK BW directly (Simon.Beuerle@bw.aok.de). In order to fulfill the legal requirements to obtain that kind of data, researchers must conclude a contract with the AOK BW regarding data access. The licensee is permitted to use the data for the purpose of the research proposal only. Licensees are not allowed to pass the data to a third party, or to create software or data bases with the exception of scientific publications. Moreover, the study has to be approved by the data protection officer both at the AOK BW and the research institute.

## Abbreviations

BW: Baden-Württemberg
DDD: Daily defined dosis
EB: Entropy Balancing
GP: General practitioner
PNP: Psychiatry-neurology-psychotherapy
PWP: Prentice, Williams and Peterson
SD: Standard deviation
UC: Usual care
