# Supplementary material for: Depression treatment in Germany – using claims data to compare a collaborative mental health care program to the general practitioner program and usual care in terms of guideline adherence and need-oriented access to psychotherapy
Source: BMC Psychiatry. 2020 Dec 14;20:591. doi: 10.1186/s12888-020-02995-1 (PMC7737360; doi:10.1186/s12888-020-02995-1)
Supplement: Supplementary file 1 — Additional file 1: Table 1a: Unadjusted statistics for the 12-month period before the index diagnosis. Table 1b: Unadjusted statistics for the 12-month period before the index diagnosis. Figure 1. Plots on treatment type, the number of therapy sessions and sick leave days for patients with mild or non-specified depression. Table 2. Estimated marginal means for sick leave days and therapy sessions in the 12-month after the index diagnosis. Table 3. Model coefficients for the number of therapy sessions in the 12-month after the index diagnosis. Figure 2. Reverse survival curves on the effect of sick leave month on the likelihood of receiving another therapy session in PNP compared to the effect in UC for the second to fifth session. Figure 3. Reverse survival curves on the effect of sick leave month on the likelihood of receiving another therapy session in PNP compared to the effect in UC for the sixth and the subsequent sessions. Figure 4. Boxplots on the number of sick leave days. Table 4. Model coefficients for sick leave days in the 12-month after the index diagnosis. Table 5. Weighted proportion of adequately or inadequately treated patients in the outpatient sector and the hospitalization rate compared between health plans in various subgroups. Table 6. Weighted proportion of adequately or inadequately treated patients by severity subgroups. [file 12888_2020_2995_MOESM1_ESM.docx]

**Supplementary Material:**

**Table 1a: Unadjusted statistics for the 12-month period before the index diagnosis**

| **Category** | **Variable** | **Control groups (pre-balancing)** | | | | **Treatment group** | |
| --- | --- | --- | --- | --- | --- | --- | --- |
|  |  | UC N=8,501 | | GP N= 4,876 | | PNP N=3,574 | |
|  |  | Mean | SD | Mean | SD | Mean | SD |
| Healthcare costs (€)  and service use | Outpatient costs | 574.91* | 594.39 | 603.51* | 532.04 | 946.24 | 704.42 |
|  | Hospital costs | 357.60* | 1490.62 | 304.22* | 1362.14 | 454.66 | 1690.92 |
|  | Costs for pharmaceuticals | 358.20* | 1283.63 | 357.01* | 1157.81 | 547.95 | 1382.93 |
|  | Sick pay | 97.46* | 818.1 | 98.49* | 794.25 | 190.75 | 1166.19 |
|  | Sick leave days | 22.04* | 33.07 | 23.92* | 33.19 | 31.33 | 41.88 |
|  | Hospital days | 0.87 | 5.79 | 0.66* | 4.23 | 1.06 | 6.50 |
| Severity of depression | First moderate episode | 0.33 | 0.47 | 0.39* | 0.49 | 0.35 | 0.48 |
|  | Recurrent moderate episode | 0.27* | 0.44 | 0.29* | 0.46 | 0.32 | 0.47 |
|  | First severe episode | 0.17* | 0.37 | 0.13 | 0.33 | 0.12 | 0.32 |
|  | Recurrent severe episode | 0.24 | 0.42 | 0.19* | 0.39 | 0.22 | 0.41 |
| Socio-demographic variables | age | 43.10* | 12.43 | 43.89* | 12.13 | 47.58 | 11.19 |
|  | male | 0.40 | 0.49 | 0.41 | 0.49 | 0.39 | 0.49 |
|  | Residency in rural area | 0.14 | 0.35 | 0.19* | 0.39 | 0.16 | 0.36 |
|  | Residency in urban area | 0.67* | 0.47 | 0.69 | 0.46 | 0.70 | 0.46 |
|  | Residency in major city | 0.19* | 0.39 | 0.12* | 0.33 | 0.14 | 0.35 |
| Quarter of index diagnosis | First quarter | 0.25* | 0.43 | 0.25* | 0.43 | 0.22 | 0.41 |
|  | Second quarter | 0.24 | 0.43 | 0.25 | 0.43 | 0.24 | 0.43 |
|  | Third quarter | 0.25 | 0.43 | 0.25 | 0.43 | 0.26 | 0.44 |
|  | Fourth quarter | 0.26* | 0.44 | 0.25* | 0.43 | 0.28 | 0.45 |
| Elixhauser (ICD-10) (physiological) | Alcohol abuse^.^ | 0.04 | 0.20 | 0.04 | 0.20 | 0.04 | 0.19 |
|  | Drug abuse | 0.02 | 0.14 | 0.02 | 0.13 | 0.02 | 0.13 |
|  | Psychosis | 0.02 | 0.14 | 0.02* | 0.12 | 0.02 | 0.15 |
|  | Depression | 0.91 | 0.28 | 0.91 | 0.29 | 0.92 | 0.27 |
|  | Anxiety disorders | 0.16* | 0.37 | 0.14* | 0.35 | 0.19 | 0.39 |
|  | Somatoform disorder | 0.22* | 0.41 | 0.19* | 0.39 | 0.24 | 0.43 |

GP general practitioner program, PNP psychiatry-neurology-psychotherapy program, UC usual care. N=16,951. The sample includes all patients that received either a moderate or severe diagnosis, statistics were rounded to 2 decimal places and calculated for the 365 day period before the sick leave diagnosis in 2015. For all variables with binary coding, 1 indicates that the condition or status was present, * indicates that the mean or proportion of the control group (UC or GP) is significantly different from the PNP group at p < .01 (calculated via t-test for independent samples or z-test)

| **Table 1b: Unadjusted statistics for the 12-month period before the index diagnosis** | | | | | | | |
| --- | --- | --- | --- | --- | --- | --- | --- |
| **Category** | **Variable** | **Control groups (pre-balancing)** | | | | **Treatment group** | |
|  |  | UC N=8,501 | | GP N= 4,876 | | PNP N=3,574 | |
|  |  | Mean | SD | Mean | SD | Mean | SD |
| Elixhauser (ICD-10) (physiological) | Congestive heart failure | 0.02* | 0.13 | 0.02* | 0.15 | 0.06 | 0.24 |
|  | Cardiac arrest | 0.06* | 0.23 | 0.05* | 0.22 | 0.08 | 0.28 |
|  | Valvular disease | 0.15* | 0.35 | 0.15* | 0.36 | 0.24 | 0,43 |
|  | Pulmonary circulation disorders | 0.00 | 0.06 | 0.00* | 0.05 | 0.01 | 0.07 |
|  | Peripheral vascular disorders | 0.02* | 0.13 | 0.02* | 0.14 | 0.04 | 0.19 |
|  | Hypertension, complicated | 0.22* | 0.42 | 0.25* | 0.43 | 0.35 | 0.48 |
|  | Hypertension, uncomplicated | 0.02* | 0.15 | 0.05* | 0.21 | 0.09 | 0.29 |
|  | Paralysis | 0.01 | 0.09 | 0.01 | 0.09 | 0.01 | 0.09 |
|  | Neurodegenerative disorders | 0.02 | 0.14 | 0.02 | 0.14 | 0.02 | 0.15 |
|  | Chronic pulmonary disease | 0.19* | 0.39 | 0.21* | 0.41 | 0.25 | 0.44 |
|  | Diabetes, uncomplicated | 0.06* | 0.24 | 0.07* | 0.26 | 0.11 | 0.31 |
|  | Diabetes, complicated | 0.02* | 0.15 | 0.04* | 0.21 | 0.08 | 0.27 |
|  | Hypothyroidism | 0.26* | 0.44 | 0.28* | 0.45 | 0.37 | 0.48 |
|  | Renal failure | 0.01* | 0.11 | 0.02* | 0.14 | 0.03 | 0.17 |
|  | Liver disease | 0.06* | 0.24 | 0.06* | 0.24 | 0.10 | 0.29 |
|  | Peptic ulcer excl. bleeding | 0.01 | 0.08 | 0.01 | 0.08 | 0.01 | 0.10 |
|  | Lymphoma | 0.00 | 0.06 | 0.00 | 0.05 | 0.00 | 0.06 |
|  | Metastatic cancer | 0.01 | 0.07 | 0.00 | 0.06 | 0.01 | 0.08 |
|  | Solid tumour without metastasis | 0.04* | 0.19 | 0.04* | 0.19 | 0.05 | 0.23 |
|  | Rheumatoid arthritis/CVD | 0.03* | 0.18 | 0.04* | 0.19 | 0.06 | 0.24 |
|  | Coagulopathy | 0.01* | 0.10 | 0.01 | 0.11 | 0.02 | 0.13 |
|  | Obesity | 0.12* | 0.32 | 0.13* | 0.34 | 0.18 | 0.38 |
|  | Weight loss | 0.02* | 0.13 | 0.01 | 0.12 | 0.01 | 0.11 |
|  | Fluid and electrolyte disorders | 0.02 | 0.14 | 0.02 | 0.13 | 0.02 | 0.15 |
|  | Blood loss anemia | 0.00 | 0.06 | 0.01 | 0.07 | 0.00 | 0.06 |
|  | Deficiency anemia | 0.04 | 0.19 | 0.04 | 0.19 | 0.05 | 0.21 |

GP general practitioner program, PNP psychiatry-neurology-psychotherapy program, UC usual care. N=16,951. The sample includes all patients that received either a moderate or severe diagnosis, statistics were rounded to 2 decimal places and calculated for the 365 day period before the sick leave diagnosis in 2015. For all variables with binary coding, 1 indicates that the condition or status was present, * indicates that the mean or proportion of the control group (UC or GP) is significantly different from the PNP group at p < .01 (calculated via t-test for independent samples or z-test)

**Figure 1: Plots on treatment type, the number of therapy sessions and sick leave days for patients with mild or non-specified depression**


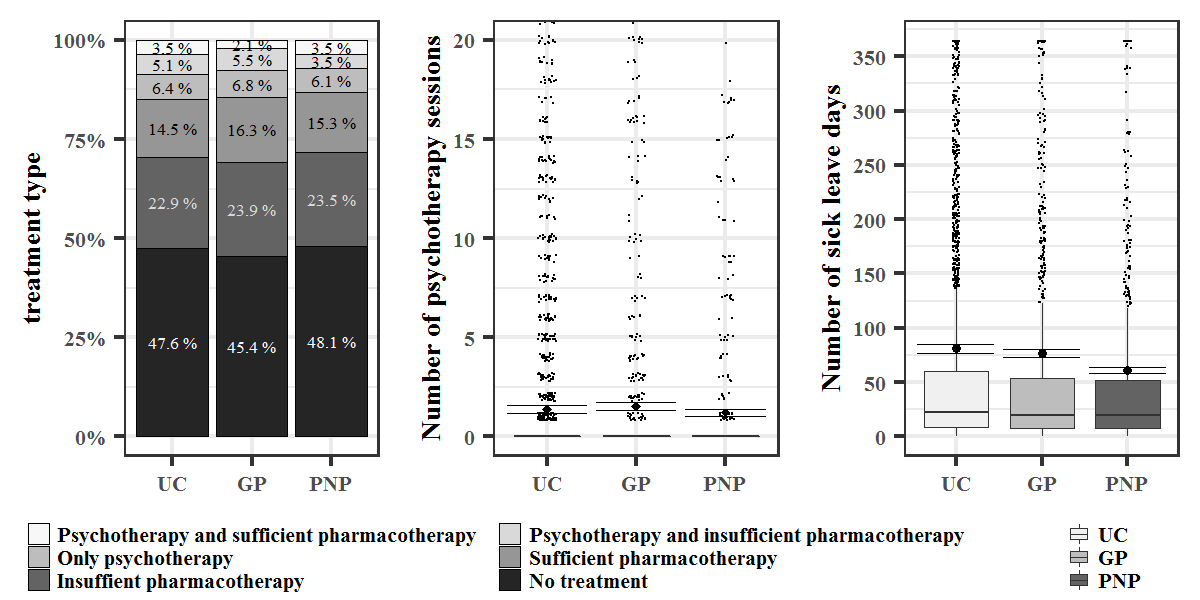


Fig. 1 GP general practitione program, PNP psychiatry-neurology-psychotherapy program, UC usual care. The results on all relevant outcomes for the residual subgroup of the N=6,294 patients classified as mild or non-specified. On the left a stacked barplot on treatment type is displayed. In the middle boxplots on the number of psychotherapy sessions are displayed. We plotted the estimated marginal means and confidence bands (± 1 Standarderror) from the two-part model. On the right we plotted the distribution of the number of sick leave days within the 12-month postperiod and added estimates from the censored negative binomial model.

| **Table 2: Estimated marginal means for sick leave days and therapy sessions in the 12-month after the index diagnosis** | | | | | | | | | | | |
| --- | --- | --- | --- | --- | --- | --- | --- | --- | --- | --- | --- |
|  |  | **Unspecified/Mild** | | **Moderate** | | | | **Severe** | | | |
|  |  |  |  | **Acute** | | **Recurrent** | | **Acute** | | **Recurrent** | |
|  |  | Estimate | Se | Estimate | Se | Estimate | Se | Estimate | Se | Estimate | Se |
| Sick leave  (post-hoc) | PNP | 60.92 | (3.01) | 83.10 | (3.38) | 107.86 | (4.47) | 146.25 | (9.81) | 169.70 | (8.10) |
|  | GP | 76.71 | (3.85) | 97.18 | (4.00) | 106.15 | (4.42) | 123.19 | (8.10) | 184.87 | (8.99) |
|  | UC | 80.96 | (4.01) | 120.78 | (5.04) | 116.28 | (4.85) | 158.12 | (10.59) | 173.69 | (8.39) |
| Therapy sessions | PNP | 1.20 | (0.18) | 3.48 | (0.24) | 5.05 | (0.29) | 2.92 | (0.36) | 5.59 | (0.36) |
|  | GP | 1.52 | (0.22) | 3.29 | (0.23) | 4.49 | (0.27) | 3.42 | (0.42) | 6.30 | (0.40) |
|  | UC | 1.39 | (0.19) | 3.73 | (0.24) | 5.26 | (0.30) | 3.29 | (0.40) | 5.64 | (0.37) |
| GP general practitioners, PNP psychiatry-neurology-psychotherapy program, UC usual care, Se standard error. The table shows the estimated marginal means for sick leave days in the 12-month postperiod from the censored-negative binomial model of the post-hoc analysis. Moreover, it shows the estimated marginal means for the number of therapy sessions within the postperiod. A session was defined as a service that requires 50 minute interaction with a licensed psychotherapists and the estimates are based on the results of the two-part model reported in supplemental table 3. | | | | | | | | | | | |

| **Table 3: Model coefficients for the number of therapy sessions in the 12-month after the index diagnosis** | | | | | | | | | | | | | | | |
| --- | --- | --- | --- | --- | --- | --- | --- | --- | --- | --- | --- | --- | --- | --- | --- |
|  | **Unspecified/Mild** | | | **Moderate** | | | | | | **Severe** | | | | | |
|  |  | | | **Acute** | | | **Recurrent** | | | **Acute** | | | **Recurrent** | | |
|  | Estimate |  | Se | Estimate |  | Se | Estimate |  | Se | Estimate |  | Se | Estimate |  | Se |
| intercept (UC) | -1.73 | *** | (0.10) | -0.79 | *** | (0.06) | -0.38 | *** | (0.06) | -0.69 | *** | (0.10) | -0.23 | ** | (0.07) |
| PNP vs. UC | -0.16 |  | (0.14) | -0.15 |  | (0.09) | -0.03 |  | (0.09) | -0.07 |  | (0.15) | 0.06 |  | (0.10) |
| GP vs. UC | -0.05 |  | (0.14) | -0.19 | * | (0.09) | -0.14 |  | (0.09) | -0.08 |  | (0.15) | 0.09 |  | (0.10) |
| Intercept (UC) | 2.23 | *** | (0.11) | 2.48 | *** | (0.05) | 2.56 | *** | (0.04) | 2.29 | *** | (0.10) | 2.54 | *** | (0.05) |
| PNP vs. UC | -0.01 |  | (0.16) | 0.04 |  | (0.07) | -0.02 |  | (0.06) | -0.07 |  | (0.14) | -0.04 |  | (0.07) |
| GP vs. UC | 0.14 |  | (0.15) | 0.01 |  | (0.07) | -0.08 |  | (0.06) | 0.09 |  | (0.14) | 0.06 |  | (0.07) |
| kappa | 1.35 | *** | (0.19) | 0.86 | *** | (0.06) | 0.78 | *** | (0.04) | 1.21 | *** | (0.15) | 0.84 | *** | (0.05) |
| GP general practitioners, PNP psychiatry-neurology-psychotherapy program, UC usual care, Se standard error. The table shows the estimated model coefficients of the two-part model that was used to model the number of therapy sessions in the 12-month postperiod. The model incorporates the weights from Entropy Balancing for the individual subgroup via weighted maximum likelihood estimation. The rows 1-3 report the results of the binomial part of the model. The rows 4-7 report the results on the truncated negative binomial part of the model. | | | | | | | | | | | | | | | |

**Figure 2: Reverse survival curves on the effect of sick leave month on the likelihood of receiving another therapy session in PNP compared to the effect in UC for the second to fifth session**


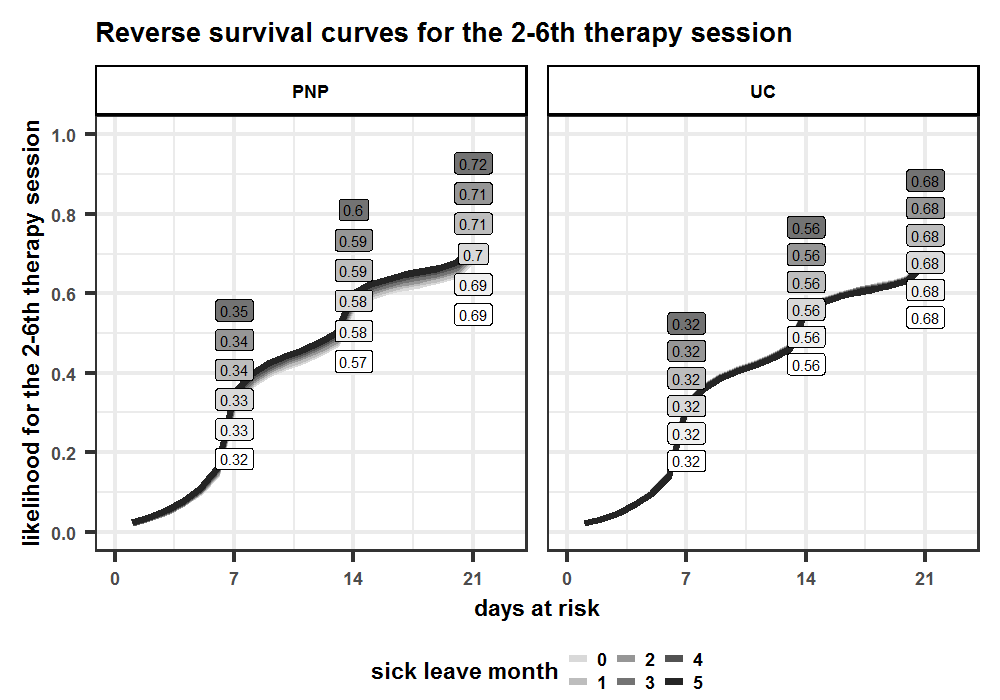


Fig. 2 PNP psychiatry-neurology-psychotherapy program, UC usual care. We predicted the likelihood for patients to receive another therapy session after they received their first to fifth session. Separate curves were predicted for patients that were on sick leave 1, 2, 3, 4 or 5 month for each health plan based on the coefficients of the PWP-gap time model. The labels show the estimated likelihood of receiving another psychotherapy session within 7, 14 and 21 days. The curves were plotted only for the comparison of usual care (UC) with the PNP program.

**Figure 3: Reverse survival curves on the effect of sick leave month on the likelihood of receiving another therapy session in PNP compared to the effect in UC for the sixth and the subsequent sessions**


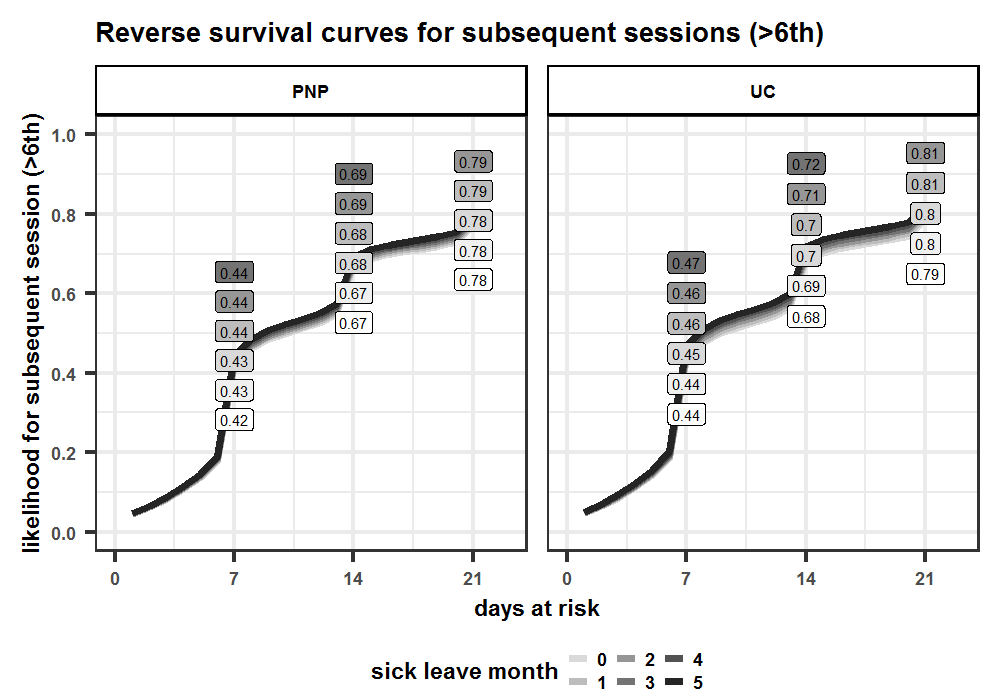


Fig. 3 PNP psychiatry-neurology-psychotherapy program, UC usual care. We predicted the likelihood for patients to receive another therapy session after they received their first six session. Separate curves were predicted for patients that were on sick leave 1, 2, 3, 4 or 5 month for each health plan based on the coefficients of the PWP-gap time model. The labels show the estimated likelihood of receiving another psychotherapy session within 7, 14 and 21 days. The curves were plotted only for the comparison of usual care (UC) with the PNP program.

**Post-hoc analysis**

**Figure 4: Boxplots on the number of sick leave days**


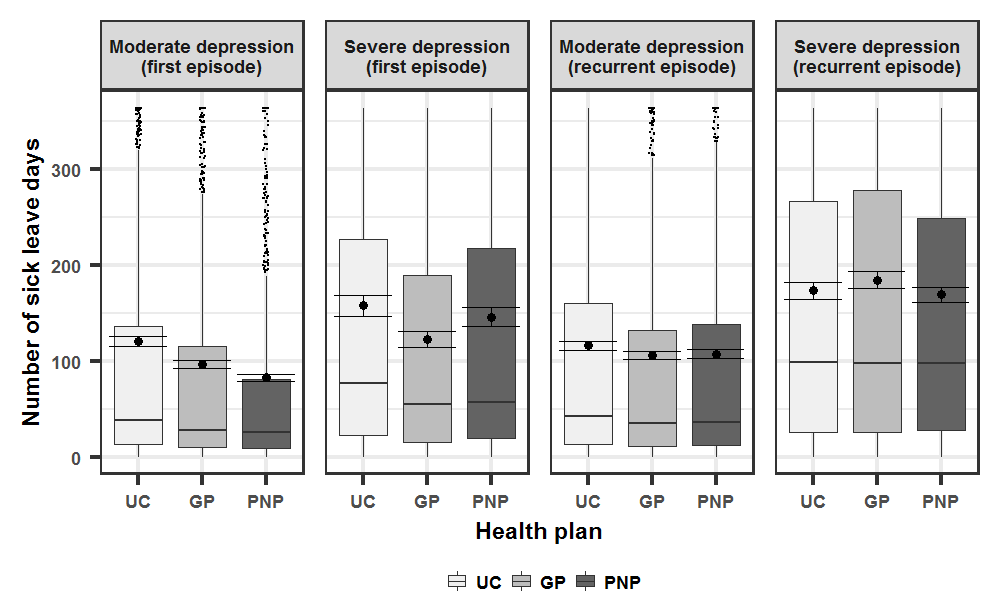


Fig. 4 Boxplots on the number of sick leave days due to common mental disorders in the 12-month observational period. We plotted the estimated marginal means and confidence bands (± 1 Standarderror) from the censored, negative binomial model reported in table 4. GP general practitioners program,

PNP psychiatry-neurology-psychotherapy program, UC usual care. N=5.918 had a first episode and N=4.879 a recurrent episode with a moderate severity. N=2.458 had a first episode and N=4.879 a recurrent episode with severe depression. For patients that received multiple diagnoses with different severities, we assigned the diagnosis with the highest severity (assuming F33 > F32 and severe > moderate > light).

Figure 4 illustrates how sick leave days due to common mental disorders were distributed in all subgroups. We added estimates from the censored (at 365 days), negative binomial model to compare the marginal means. For patients with moderate depression, the average number of sick leave days was 37.68 days lower in the PNP program than in UC, t(5918)=-6.21, p<.001, and 14.08 days lower than in the GP program, t(5918)=-2.69, p=.0071. Thus, the GP program also had on average 23.59 fewer sick leave days than UC, t(5918)=3.67, p=.0002. For patients with a first episode of severe depression, we found that patients in the GP program had on average 34.94 fewer sick leave days than patients in UC, t(2458)=-2.63, p=.0087, and descriptively 23.06 fewer than in the PNP program, t(2458)=-1.82, p=.0693. For the patients with recurrent depression, we observed no significant differences in sick leave days between health plans – irrespective of severity.

| **Table 4: Model coefficients for sick leave days in the 12-month after the index diagnosis** | | | | | | | | | | | | | | | |
| --- | --- | --- | --- | --- | --- | --- | --- | --- | --- | --- | --- | --- | --- | --- | --- |
|  | **Unspecified/Mild** | | | **Moderate** | | | | | | **Severe** | | | | | |
|  |  | | | **Acute** | | | **Recurrent** | | | **Acute** | | | **Recurrent** | | |
|  | Estimate |  | Se | Estimate |  | Se | Estimate |  | Se | Estimate |  | Se | Estimate |  | Se |
| intercept (UC) | 4.39 | *** | (0.05) | 4.79 | *** | (0.04) | 4.76 | *** | (0.04) | 5.06 | *** | (0.07) | 5.16 | *** | (0.05) |
| PNP vs. UC | -0.28 | *** | (0.07) | -0.37 | *** | (0.06) | -0.08 |  | (0.06) | -0.08 |  | (0.09) | -0.02 |  | (0.07) |
| GP vs. UC | -0.05 |  | (0.07) | -0.22 | *** | (0.06) | -0.09 |  | (0.06) | -0.25 | ** | (0.09) | 0.06 |  | (0.07) |
| kappa | 1.88 | *** | (0.05) | 1.83 | *** | (0.04) | 1.74 | *** | (0.04) | 1.59 | *** | (0.06) | 1.47 | *** | (0.04) |
| GP general practitioners, PNP psychiatry-neurology-psychotherapy program, UC usual care, Se standard error. The table reports the model coefficients of the censored (at 365 days) negative binomial model. The model incorporates the weights from Entropy Balancing for the individual subgroup via weighted maximum likelihood estimation. | | | | | | | | | | | | | | | |

| **Table 5: Weighted proportion of adequately or inadequately treated patients in the outpatient sector and the hospitalization rate compared between health plans in various subgroups** | | | | | | | | | | | | | | | |
| --- | --- | --- | --- | --- | --- | --- | --- | --- | --- | --- | --- | --- | --- | --- | --- |
|  | **Unspecified/Mild** | | | **Moderate** | | | | | | **Severe** | | | | | |
|  |  |  |  | **Acute** | | | **Recurrent** | | | **Acute** | | | **Recurrent** | | |
| Health plan status | UC | PNP | GP | UC | PNP | GP | UC | PNP | GP | UC | PNP | GP | UC | PNP | GP |
| Sample size | 3870 | 831 | 1593 | 2781 | 1236 | 1901 | 2300 | 1146 | 1433 | 1415 | 423 | 620 | 2005 | 769 | 922 |
| adequate AD treatment and ≥10 sessions | 1.6% | 1.9% | 1.2% | 4.8% | 4.7% | 3.9% | 9.8% | 8.6% | 6.6%* | 6.2% | 5.2% | 4.8% | 12% | 12.1% | 14.5% |
| ≥10 sessions | 3.1% | 2.0% | 3.6% | 6.2% | 5.6% | 6.0% | 6.3% | 7.2% | 5.9% | 3.4% | 3.3% | 2.6% | 2.7% | 4.0% | 5.1%* |
| adequate AD treatment | 14.5% | 15.3% | 16.3% | 15.0% | 13.0% | 13.7% | 18.7% | 16.8% | 17.6% | 18.7% | 19.1% | 19.2% | 24.2% | 21.8% | 22.7% |
| No adequate treatment | 80.8% | 80.7% | 78.9% | 74.0% | 76.7% | 76.4% | 65.2% | 67.4% | 69.8%* | 71.7% | 72.3% | 73.3% | 61.1% | 62.0% | 57.7% |
| Hospitalization rate | 0.2 % | 0.2 % | 0.1 % | 3.8 % | 3.1 % | 2.6 % | 7.7 % | 7.1 % | 5.7%* | 17.7% | 13.2%* | 16.1% | 33.7% | 30.8% | 33.2% |
| GP general practitioners, PNP psychiatry-neurology-psychotherapy program, UC usual care, AD antidepressant, sessions refer to psychotherapy services that require 50 minute interactions with a licensed psychotherapists. We calculated the chi-square test with the Rao-Scott second order correction, in order to compare the proportion of the different treatment types in the three health plans, *p<.05. The definition of adequate AD treatment is provided in table 2 of the orginal article. All proportions were adjusted with the weights obtained from Entropy Balancing for the individual subgroup | | | | | | | | | | | | | | | |

| **Table 6: Weighted proportion of adequately or inadequately treated patients by severity subgroups** | | | | | |
| --- | --- | --- | --- | --- | --- |
|  | **Unspecified/Mild** | **Moderate** | | **Severe** | |
|  |  | **Acute** | **Recurrent** | **Acute** | **Recurrent** |
| Sample size | 6294 | 5918 | 4879 | 2458 | 3696 |
| adequate AD treatment and ≥10 sessions | 15.4% | 13.9% | 17.7% | 19% | 22.9% |
| ≥10 sessions | 1.6% | 4.5% | 8.3% | 5.4% | 12.9% |
| adequate AD treatment | 2.9% | 5.9% | 6.5% | 3.1% | 3.9% |
| No adequate treatment | 80.2% | 75.7% | 67.5% | 72.4% | 60.3% |
| AD antidepressant, sessions refer to psychotherapy services that require 50 minute interactions with a licensed psychotherapists. The definition of adequate AD treatment is provided in table 2 of the orginal article. All proportions were adjusted with the weights obtained from Entropy Balancing for the individual subgroup | | | | | |
